# Zoledronic acid targets chemo-resistant polyploid giant cancer cells

**DOI:** 10.1038/s41598-022-27090-1

**Published:** 2023-01-09

**Authors:** Rezvan Adibi, Shiva Moein, Yousof Gheisari

**Affiliations:** 1 Department of Genetics and Molecular Biology, Isfahan University of Medical Sciences, Isfahan, Iran; 2 Regenerative Medicine Research Center, Isfahan University of Medical Sciences, Isfahan, 8174673461 Iran

**Keywords:** Cancer metabolism, Cancer, Cell biology

## Abstract

Although polyploid giant cancer cells (PGCCs) are known as a key source of failure of current therapies, sufficient drugs to target these cells are not yet introduced. Considering the similarities of polyploid cells in regeneration and cancer, we hypothesized that zoledronic acid (ZA), an osteoclast-targeting agent, might be used to eliminate PGCCs. The 5637-bladder cancer cell line was treated with various doses of cisplatin to enrich polyploid cells and the efficacy of different concentrations of ZA in reducing this population was assessed. The metabolic profile of PGCCs was investigated with gas chromatography-mass spectrometry. Lipid profiles, mitochondrial density, and ROS content were also measured to assess the response of the cells to ZA. Cancer cells surviving after three days of exposure with 6 μM cisplatin were mainly polyploid. These cells demonstrated special morphological features such as fusion with diploid or other polyploid cells and originated in daughter cells through budding. ZA could substantially eradicate PGCCs with the maximal effect observed with 50 μM which resulted in the drop of PGCC fraction from 60 ± 7.5 to 19 ± 1.7%. Enriched PGCCs after cisplatin-treatment demonstrated a drastic metabolic shift compared to untreated cancer cells with an augmentation of lipids. Further assays confirmed the high content of lipid droplets and cholesterol in these cells which were reduced after ZA administration. Additionally, the mitochondrial density and ROS increased in PGCCs both of which declined in response to ZA. Taken together, we propose that ZA is a potent inhibitor of PGCCs which alters the metabolism of PGCCs. Although this drug has been successfully exploited as adjuvant therapy for some malignancies, the current evidence on its effects on PGCCs justifies further trials to assess its potency for improving the success of current therapies for tackling tumor resistance and relapse.

## Introduction

Cancer is the second leading cause of death and one of the most problematic health issues in the world. Despite achievements in the establishment of treatment strategies that halt cancer progression, tumor relapse post-chemo/radiotherapy remains to be the bottleneck of cancer treatment. Understanding the mechanisms of cancer recurrence would be beneficial in designing more efficient treatments. Among various mechanisms supposed to have a role in tumor relapse, multinucleation is so prevalent and thought to be one of the main contributors. The first sketches of genome multiplication were drawn by Virchow in around 2 centuries ago^1,2^. Afterward in 1902, Boveri suggested a link between genome doubling and the appearance of aneuploidy in cancer cells^3^. Since then, the presence of polyploid cells in different types of cancers and their augmentation upon chemo/radiotherapy is reported frequently^4–6^.

The therapy-resistant polyploid cells can reconstitute the tumor tissue by the production of mononucleated cells through budding or bursting^7^. Interestingly, it is recently shown that single PGCCs can form spheroids that are indistinguishable from human blastomeres. Furthermore, PGCCs showed activation of stem cell markers and were able to differentiate to all three germ layers, suggesting the employment of evolutionarily conserved mechanisms by PGCCs for cancer progression^8^. The time- and context-dependent expression of cancer stem cell markers such as OCT4, NANOG, SOX2, and SSEA1 by PGCCs is also reported by other investigators^2,9–11^. Notably, it is shown that the transplantation of a single PGCC could regenerate the entire tumor cells with the ability of metastasis to lung^12^. Furthermore, polyploidization/depolyploidization cycles are error-prone resulting in genomic instability and augmented heterogeneity in the tumor microenvironment^13–16^. This increased diversity could be beneficial for cancer cell survival in response to different kinds of stressors naturally present in the tumor microenvironment or insults from therapeutic agents^9,17,18^.

Although the role of PGCCs in cancer progression and relapse is well recognized, clinically validated drugs that could efficiently target these cells are only introduced in a few studies; the mTOR inhibitor, rapamycin, induces autophagy and apoptosis in PGCCs^19,20^. Also, inhibition of glycolysis by 2-deoxy-d-glucose led to the decrement of PGCCs^19,21^. Targeting apoptotic pathways^22^, MAPK signaling^23^, and cell cycle regulators^24^ are also proposed to be effective in eliminating PGCCs. However, these introduced strategies are suboptimal, highlighting the importance of designing more efficient therapeutics to eradicate these roots of cancer.

Considering the accumulating evidence on the presence of polyploidy in normal cells of different organs^25^, we have recently discussed the idea that polyploidy is an evolutionarily conserved mechanism hijacked by cancer cells^7^. Taking into account the similarity of osteoclasts and PGCCs^26,27^, we hypothesized that drugs targeting osteoclasts could be repositioned against PGCCs. Hence, we designed this study to investigate the effect of Zoledronic Acid (ZA), a potent inhibitor of osteoclasts, on PGCCs. This bisphosphonate drug is already in the market for osteoporosis with an acceptable safety record^28,29^. It is also used for the management of dysregulated bone remodeling in Paget’s disease and bone-destructive lesions in multiple myeloma^30^. ZA inhibits metabolism and induces apoptosis of osteoclasts. It also interferes with the attachment of osteoclasts to bone matrix. In addition to anti-bone resorption properties, it is demonstrated that the combinatorial use of ZA and chemotherapy agents has beneficial effects in the treatment of breast, bladder, kidney, and lung cancers^31–34^, which is supposed to be mediated through different mechanisms such as inhibition of tumor-associated macrophages and declining angiogenesis^35–37^. However, to our knowledge, the inhibition of PGCCs by bisphosphonates is not yet described.

In this study, we have shown that ZA has a significant potency in eradicating PGCCs which become enriched after cisplatin administration. We also demonstrate that PGGCs have a distinct metabolic profile and at least a part of the effect of ZA on PGCCs is exerted through alteration of cell metabolism.

## Results

### Cisplatin induces polyploidy in cancer cells

**Figure 1:**
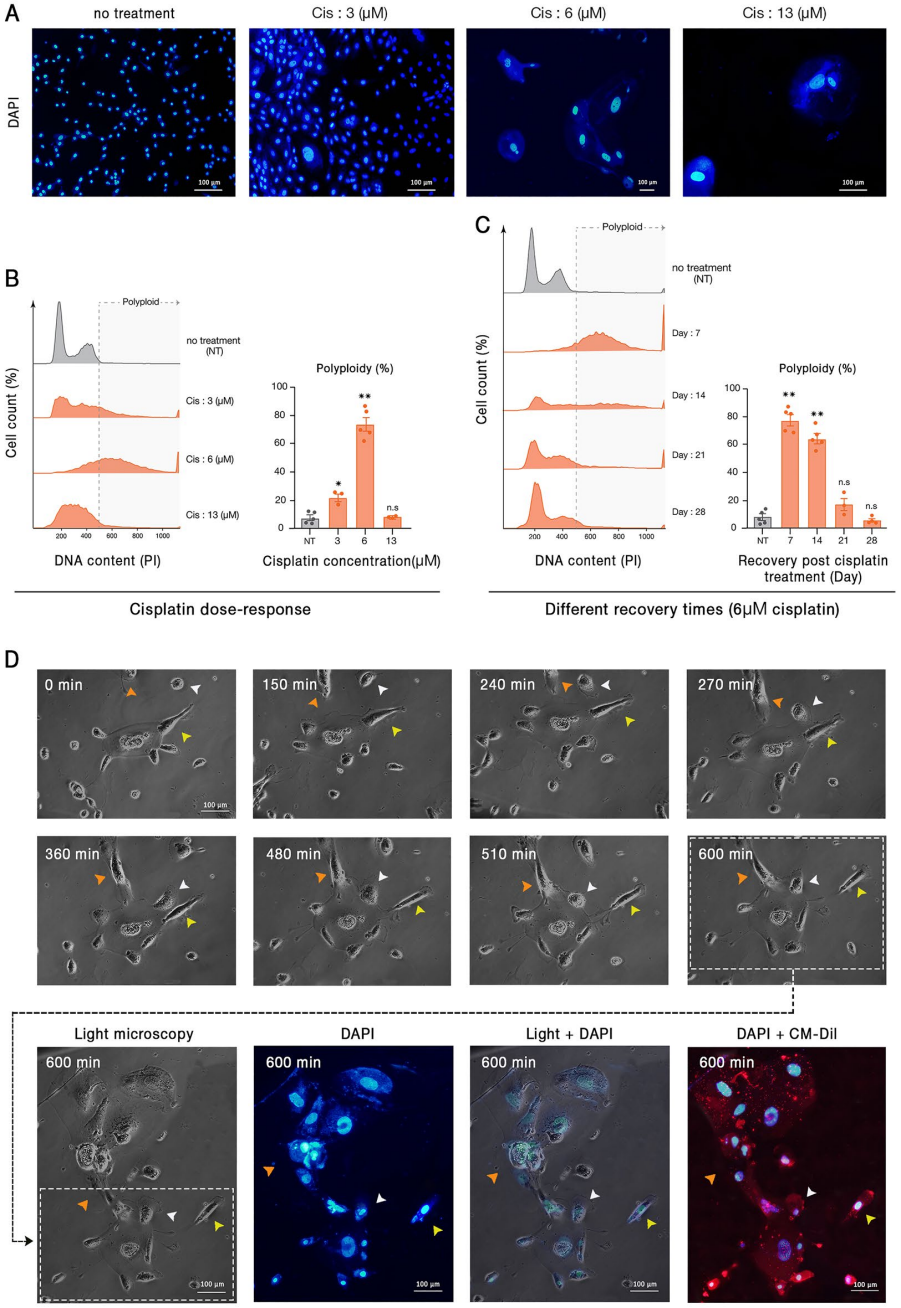
Cisplatin-induced polyploid cells demonstrate a dynamic polyploidization and depolyploidization characteristic. Cisplatin administration resulted in significant cell death and the enrichment of large polyploid cells. (**A**) Representative images of cells treated with 3, 6, and 13 μM cisplatin for 72 h are shown. In this panel, all pictures are captured with 20X objective lens except the third one (Cis 6 μM) which is taken with 10X. (**B**) DNA content analysis of treated cells with different doses of cisplatin indicated that the 6 μM cisplatin results in the highest rate of polyploidy. (**C**) Assessment of different recovery periods following the three days of 6 μM cisplatin administration revealed that polyploidy is maximal with a 7 days recovery time. (**D**) Time-lapse imaging of cisplatin-treated PGCCs demonstrated considerable changes in the genome content and cytoplasm morphology. White arrowheads: fusion with a diploid cell; orange arrowheads: fusion with another PGCC; yellow arrowheads: budding of daughter cells. Nuclei and cell membrane are stained with DAPI and CM-Dil, respectively; Cis: Cisplatin; * *p-*value < 0.05; ** *p-*value < 0.01; error bars: mean ± SEM.

Cisplatin is a vastly used chemotherapeutic agent that targets DNA and makes double-strand breaks. Several investigations including our recent study have shown the effect of cisplatin in induction of PGCCs^38,39^. In order to determine the optimal concentration of cisplatin that induces the highest proportion of polyploid cells, 5637 bladder cancer cells were treated with 3, 6, 13, 50, 75, and 100 μM of cisplatin. After 72 h treatment with cisplatin, the cells were kept in normal conditions for a recovery time of 7 days. Doses of 50, 75, and 100 μM were lethal, and after drug administration, no viable cells remained (Figs. 1A and B). However, after treatment with 3, 6, and 13 μM, a population of cells survived that were enriched for PGCCs. Based on fluorescence microscopy (Fig. 1A) and cytofluorometric DNA content analysis (Fig. 1B), 6 μM cisplatin has the maximal effect on PGCC enrichment. In addition, it is critical to note that the eradication of mononucleated cells, the augmentation of PGCCs, and the budding of mononucleated cells are time-dependent processes. Hence, we were interested in identifying the recovery time in which the highest proportion of PGCCs appears. One month follow-up of the cells treated with 6 μM cisplatin revealed that PGCCs are most augmented after a 7-days recovery period (Fig. 1C). After this time, the proportion of PGCCs declines until it reaches levels of untreated cells in weeks 3 and 4. This could be described by the genomic content reduction through the budding of mononucleated cells from PGCCs as described in the next section. Based on the above findings, in the next experiments, cells were treated with 6 μM cisplatin for 3 days and then allowed to recover for 7 days before measurements.

### PGCCs show high morphological plasticity

The morphology of PGCCs was evaluated by time-lapse light and fluorescent microscopy, showing that PGCCs are capable of producing mononucleated progenies via budding (Fig. 1D). Besides, bursting of a multinucleated cell into various mononucleated cells was observed (Figure S1). These findings are in agreement with our previous results on mesenchymal stem cells^40^. Furthermore, the fusion of a mononucleated cell with a PGCC or fusion of two PGCCs was observed frequently (Fig. 1D). The coordinated occurrence of budding and fusion indicates the high plasticity of the genome and cytoplasm of these cells which has received less attention in classical biology.

### ZA targets PGCCs enriched after cisplatin administration

**Figure 2:**
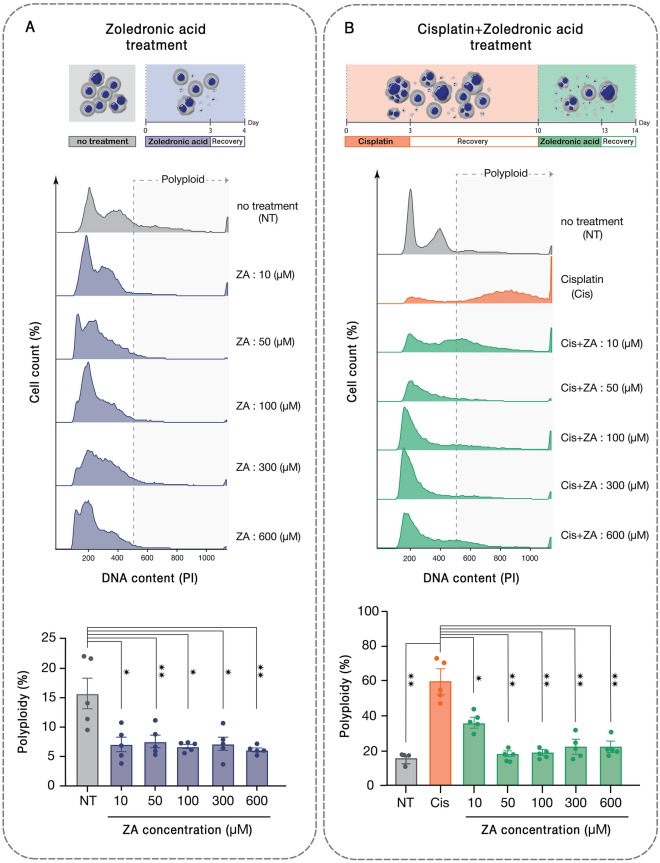
Zoledronic acid declines the population of PGCCs pre- and post-cisplatin administration. (A) DNA content analysis of 5637 bladder cancer cells treated with different doses of ZA indicates the reduction of PGCCs post-treatment. (B) Cisplatin administration enriches PGCCs and ZA at different doses from 10–600 μM declines the PGCC population with the most profound effect being observed at 50 μM. *p-value < 0.05; ** *p-* value < 0.01; error bars: mean ± SEM.

PGCCs have common properties with osteoclasts, especially in terms of genome content and it is rational to assume that similar molecular mechanisms drive these mutual phenotypes. Hence, we were interested to assess if osteoclast inhibiting agents could be exploited to target PGCCs. The effect of different doses of ZA on the polyploid sub-population of 5637 bladder cancer cell line was investigated. A three-day treatment of these cells with ZA followed by 24 h recovery resulted in a drastic decline in the polyploid fraction (Fig. 2A). PGCCs are recognized as a main source of tumor relapse following initial response to chemotherapy. To simulate this situation, the PGCC-enriched population remaining after treatment with 6 μM cisplatin was exposed to ZA. All examined doses of ZA ranging from 10 to 600 μM could substantially eradicate PGCCs with the maximal effect observed with 50 μM which resulted in the drop of PGCC fraction from 60 ± 7.5 to 19 ± 1.7% (*p *< 0.01; Fig. 2B).

Cell survival was also assessed with trypan blue staining in the four experimental groups, including control cells, treated with either cisplatin or ZA and consequential treatment with both drugs. ZA reduced cell survival from 100% to 33.7% (*p *< 0.05). After treatment with cisplatin, only 1.3% of the cells survived. This rate further declined to 0.2% when the cells were additionally treated with ZA.

(*p *< 0.05; Figure S2). Taken together, ZA not only augments the cytotoxic effect of cisplatin but also significantly decreases the ratio of polyploid to diploid sub-populations.

We were also interested to see if ZA has similar effects on the polyploid population of non-cancer cells. Hence, mouse bone marrow-derived mesenchymal stem cells and human foreskin fibroblasts were exposed to 50 μM ZA. Interestingly, about 1% of human foreskin fibroblasts (HFF) were polyploid that remained almost unresponsive to ZA. The rate of polyploidy of mesenchymal stem cells (MSCs) slightly declined from 7.4 ± 0.7% to 5.5 ± 0.6% (*p *= 0.05; Figure S3A). Furthermore, trypan blue staining was performed to assess the toxicity of this drug on normal cells which demonstrated that the viability of both HFF and MSC populations was reduced by about 50% upon treatment with 50 μm of ZA (Figure S3B).

### Lipid metabolism and mitochondria function alter in cisplatin-induced PGCCs

**Figure 3:**
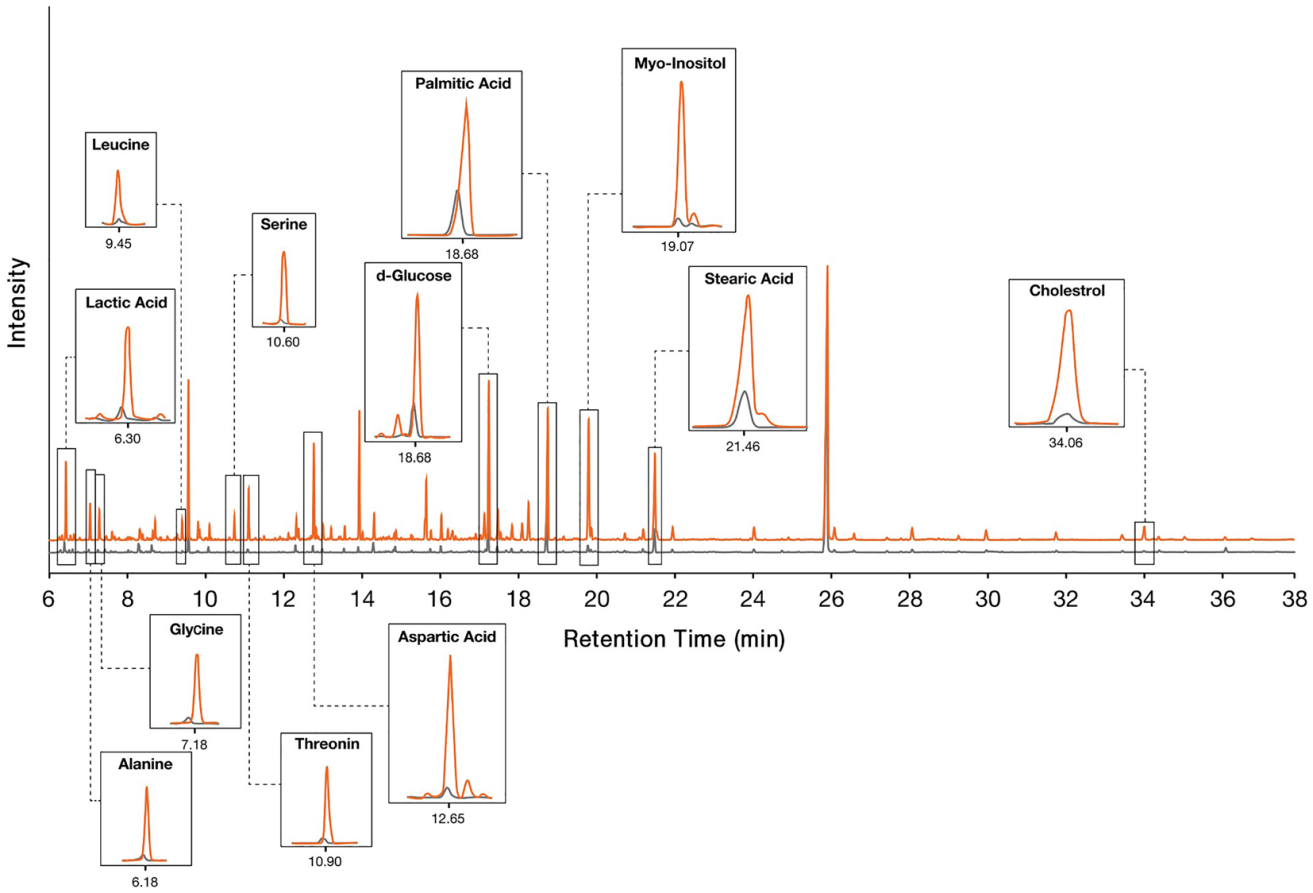
Cisplatin-treated PGCC-enriched cells have a distinct metabolic profile. The metabolome of cisplatin-treated (orange peaks) and untreated bladder cancer cells (gray peaks) were assessed by gas chromatography-mass spectrometry (GC–MS).

The cellular mechanisms underlying the unique feature of PGCCs are still largely unknown. To get an overview of these cells, GC-mass analysis was performed to inspect these cells from a metabolic point of view. The metabolic profile of cisplatin-treated cells was compared to untreated cancer cells (Fig. 3). Considerable changes in amino acids like aspartic acid, threonine, alanine, glycine, serine, and leucine were detected. Also, lactic acid and d-glucose which represent changes in cell metabolism were among the altered metabolites. In addition, lipid molecules including cholesterol, stearic acid, and palmitic acid were increased in cisplatin-treated cells.

**Figure 4:**
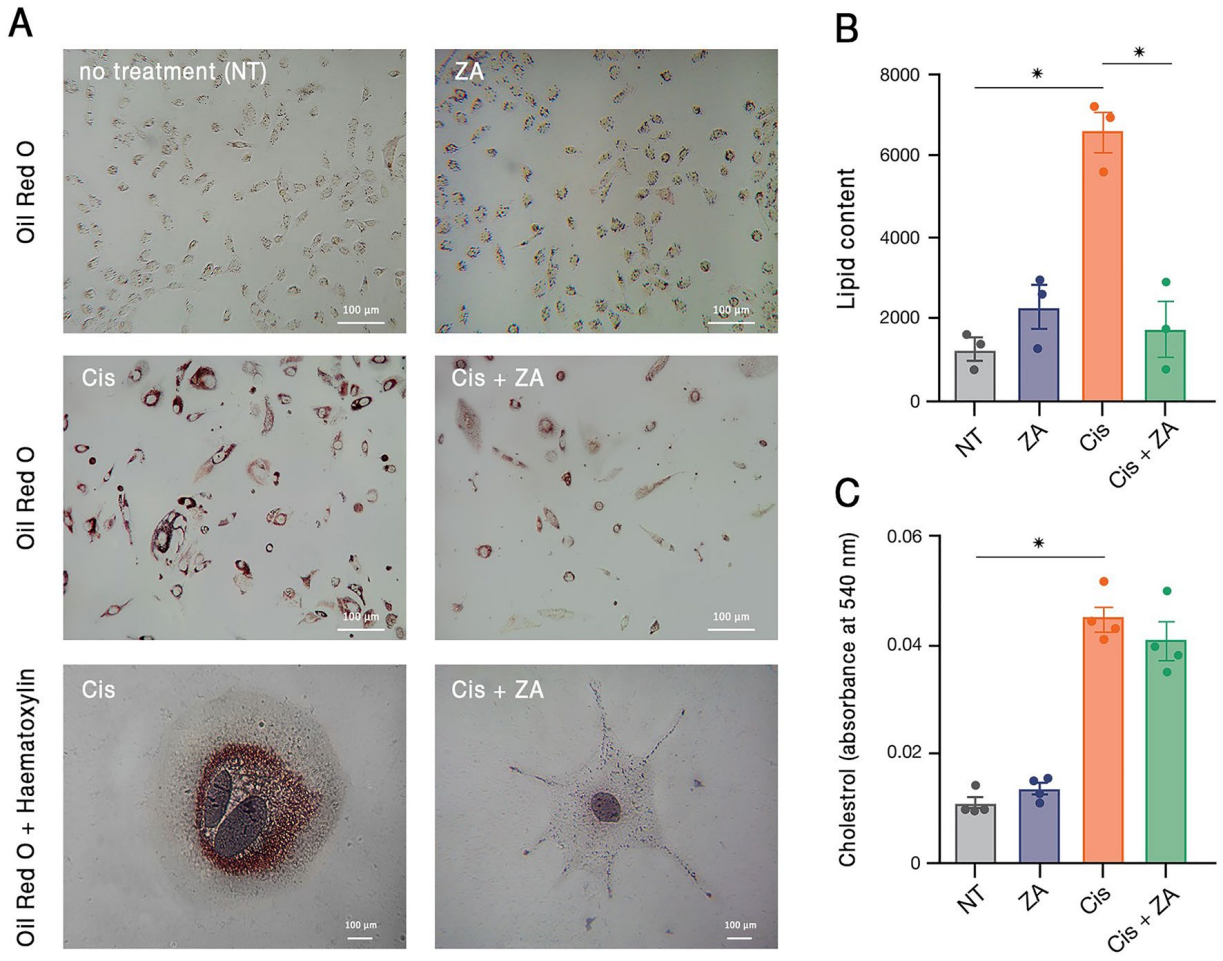
PGCCs have augmented lipid metabolism. (**A**) Oil Red O staining demonstrated the accumulation of lipid droplets in PGCCs surviving after cisplatin treatment which partly declined after ZA treatment. Nuclei are stained with haematoxylin. (**B**) Quantification of the red signals in the images of Oil Red O-stained cells in three independent experiments confirmed the microscopic observations. (**C**) Total cholesterol content of cancer cells increased after cisplatin treatment. * *p-*value < 0.05; error bars: mean ± SEM.

Considering the alteration of lipid metabolism in PGCCs, we were interested to assess the effect of ZA on the lipid profile of these cells. The prevalence of lipid droplets in PGCCs was assessed by Oil Red O staining. The untreated cells harbored a low amount of lipid droplets which drastically increased upon cisplatin administration. Interestingly, treatment with ZA, resulted in a significant decrease in the amount of these vesicles (Fig. 4A,B). To further investigate alterations in lipid metabolism, cholesterol content was measured. In correlation with Oil Red O staining data, cholesterol content considerably increased in response to cisplatin but slightly decreased after ZA treatment (Fig. 4C). A similar trajectory was observed for triglycerides (Figure S4). Taken together, it is supposed that PGCCs undergo a metabolic shift with significant changes in lipid metabolism that are efficiently targeted with ZA.

### PGCCs have a high mitochondrial density

**Figure 5:**
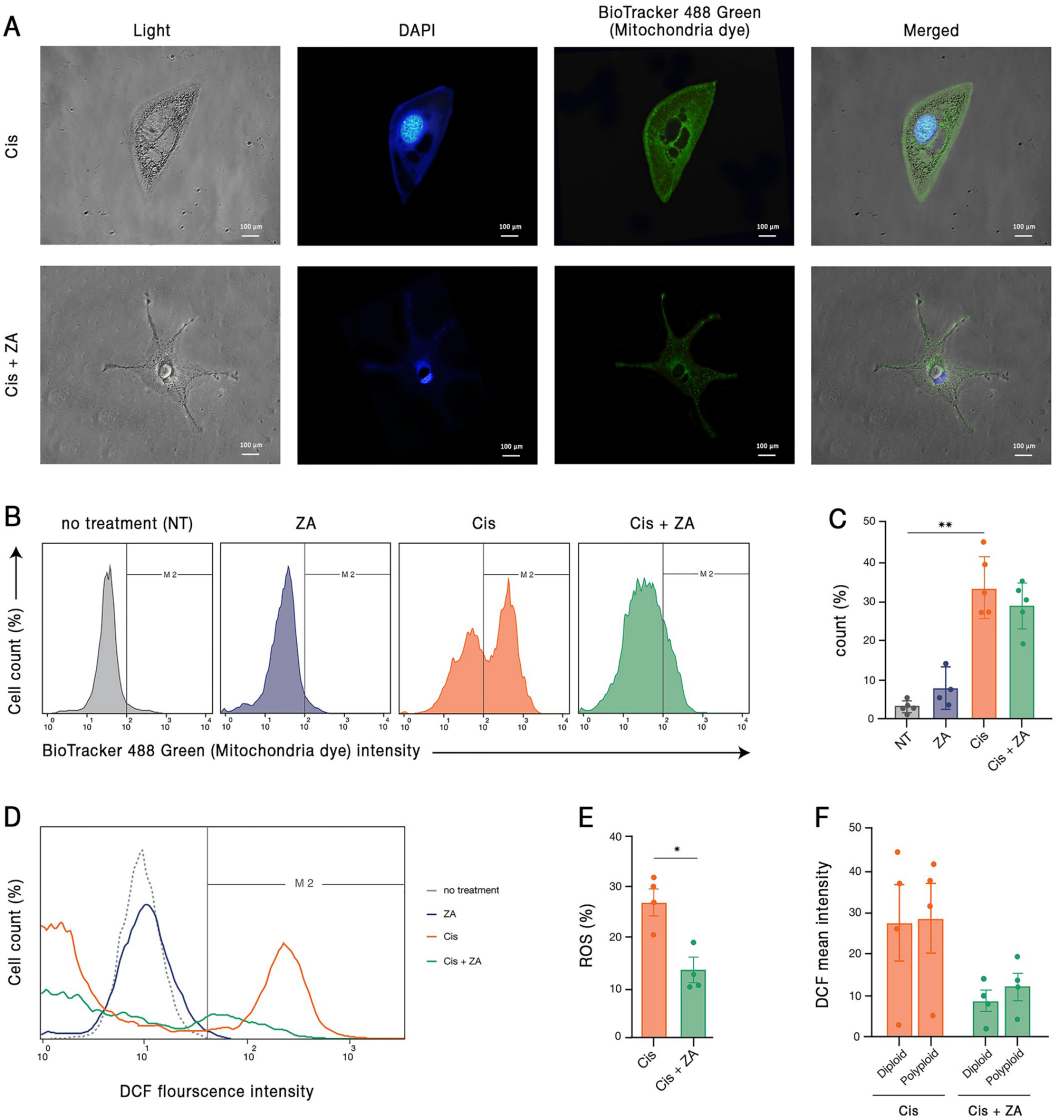
Cisplatin-surviving cells have high mitochondrial density and ROS content, both of which are reduced by ZA. (**A**) Representative images of mitochondrial staining of cancer cells treated with cisplatin or cisplatin + ZA are demonstrated. (**B**) Representative flow-cytometry graphs and (**C**) quantification of four independent experiments demonstrated the augmentation of mitochondrial density after cisplatin and the partial declining effect of ZA. (**D**, **E**) ROS content of cisplatin-survived cells is increased and is significantly diminished after ZA administration. (**F**) The effect of ZA in declining ROS in cisplatin-treated cells is observed both in diploid and polyploid sub-populations. *p- value < 0.05; ** *p-* value < 0.01; error bars: mean ± SEM.

In line with the described active cytoplasm and nuclear morphological dynamism, the significant metabolic changes observed in PGCCs suggest that energy balance is essential for the functionality of these cells. In this regard, we were interested to assess the mitochondrial content of PGCCs. The mitochondria were stained with BioTracker 488 green and the mitochondrial content was evaluated by fluorescent microscopy and flow cytometry. It was found that cisplatin administration is associated with a considerable increase in the density of mitochondria. After treatment with ZA, a mild decline is observed in the content of mitochondria (Fig. 5A–C).

As an augmentation of mitochondrial density might be correlated with incremented levels of ROS, the cells were stained with DCFDA and ROS content was assessed by flow cytometry. Although ZA alone did not have an obvious impact on ROS content, cisplatin treatment resulted in a considerable increase in ROS. This elevated level of ROS was significantly reduced upon ZA administration (Fig. 5D,E). Notably, ZA had a similar effect on ROS level of both multi and mononucleated sub-populations of cisplatin-treated cells (Fig. 5F). Taken together, regulation of mitochondria density and ROS production are essential elements of the biology of PGCCs.

### A fraction of PGCCs show cytoplasmic vacuolization

**Figure 6:**
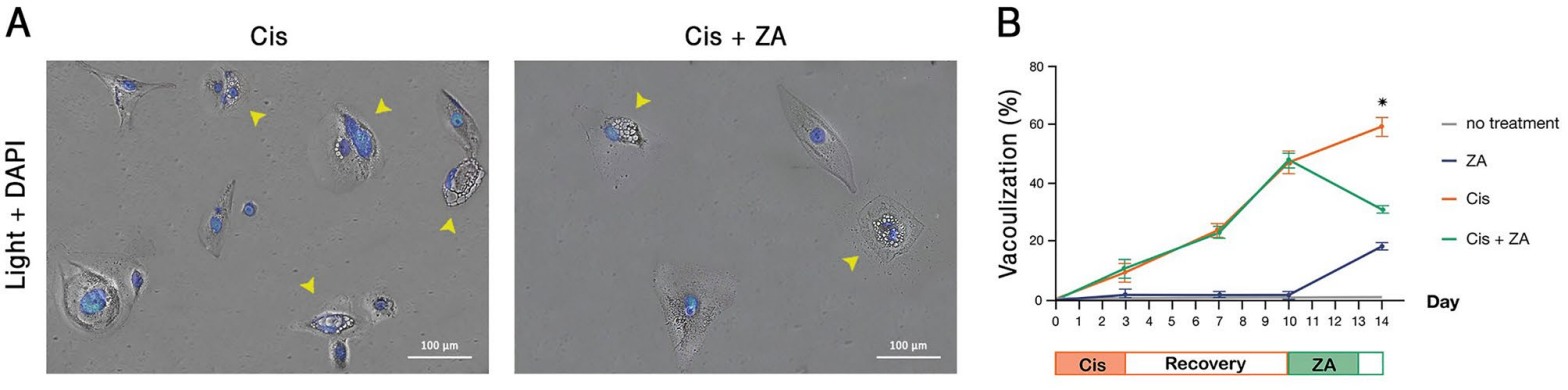
Cytoplasmic vacuolization is a common morphology in cisplatin-induced PGCCs. (**A**) Representative images of vacuolated cells (arrowheads) in cisplatin and cisplatin + ZA treated groups are shown. Nuclei are stained with DAPI. (**B**) Time-series quantification of microscopic observations in three independent experiments demonstrated the accumulation of these vacuoles in cisplatin-treated cells. The asterisk indicates the significant decline of these vacuoles in Cis + ZA group compared to Cis (*p-*value < 0.05); error bars: mean ± SEM.

An interesting observation during the microscopic inspection of PGCCs was the appearance of large vacuoles in their cytoplasm. Considerably, the number of vacuolated cells increased over time after cisplatin administration but decreased significantly post ZA treatment (Figs. 6A,B). Vacuolization is not observed in all of the PGCCs and these vacuoles are distinct from lipid droplets and nuclei (Figure S5). Although formation of vacuoles has previously been reported in response to cancer therapeutics^41^ and some investigators have attributed them to therapy resistance^42^, the content and function of these structures are yet elusive.

## Discussion

Resistance to chemotherapy and relapse are the main causes of the failure of current cancer treatments. PGCCs, the hallmarks of various tumors, are a potential origin of cancer stem cells and initiators of tumor relapse post-treatment. Nonetheless, few studies have focused on therapeutic approaches that target these cells. In this study, we found that polyploidy increases upon cisplatin administration in 5637 bladder cancer cells, and PGCCs could be the origin of mononucleated cells through budding. Furthermore, it is demonstrated that ZA, an osteoclast targeting drug, can efficiently eradicate PGCCs that become enriched after cisplatin administration.

We followed the behavior of polyploid cells in the culture. Time-lapse imaging showed that mononucleated progenies bud from PGCCs. Also, fusion of mononucleated and multinucleated cells was demonstrated. PGCCs are called “pregnant cells” capable of generating daughter cells and transferring genome content via funiculus-like structures^10^. Cell–cell fusion, budding and bursting are also reported by Zhang et.al^9^. We observed that polyploidization/depolyploidization cycles occur simultaneously in one cell. This observation indicates that not only genome amplification, compaction, and segregation processes are distinctive in these giant cells, but also cell cytoskeleton plays a critical role in maintenance of their function. Notably, these features are not restricted to PGCCs and normal polyploid cells take advantage of same mechanisms, especially in response to stressors^7,40^. Thus, decoding the molecular processes of normal polyploid cells could provide an opportunity for developing effective therapies against PGCCs.

Osteoclast and PGCCs share lots of genomic and phenotypic resemblances. The appearance of polyploid “osteoclast-like cells” has been reported in various metastatic tumor lesions^43–45^. Moreover, the involvement of osteoclasts in giant cell tumor of bone^46^ provide further supports for the similarity of these two cell types. In agreement, denosumab, a monoclonal antibody inhibiting the formation of osteoclasts through targeting an essential protein for macrophage fusion, is shown not only to be effective for osteoporosis but also for breast cancers with bone metastasis^47^. We have here shown that ZA, a bisphosphonate safely used for osteoporosis, has a significant effect on the removal of PGCCs. It has recently been observed that concomitant use of this drug with cisplatin in breast, bladder, kidney, and lung cancer has increased the efficacy of treatment (34–37). However, to date, no study has specifically examined the effect of ZA on PGCCs.

The understanding of the molecular biology of polyploid cells, both in cancer and regeneration, is just in its beginning. Although differential expression of some proteins related to cell cycle and stemness is reported in previous studies^2,10,11,48^, no specific marker of PGCCs is yet introduced. This has hindered the development of a standard protocol for the isolation and characterization of these cells. High throughput omics studies are of special value for the identification of such putative markers. Furthermore, given the complexity of these cells, the use of such holistic approaches could be useful in deciphering the molecular aspects. Our metabolomics study showed that PGCCs enriched post cisplatin administration have a different pattern of lipid profile compared to untreated cells. Oil Red O staining and cholesterol measurement verified this data and confirmed that ZA has a prominent effect on lipid metabolism. In support with this notion, lipid metabolism of cancer cells has recently received considerable attention; Siros et.al showed the importance of lipids and mitochondria in survival of the chemo-resistant cancer cells and reported that PGCCs shift their metabolism to higher dependence on fatty acids and oxidative phosphorylation^49^. The increment of lipid droplets in these cells has also been observed^49,50^. Indeed, lipids as energy stores enable cancer cells to efficiently endure long-term stress condition^51^. Inline, our unpublished data underscores the role of lipid metabolism in the function of bone marrow regenerative polyploid cells. Hence, a shift in lipid metabolism seems to be a common property of both PGCCs and normal polyploid cells.

Microscopic inspections determined active dynamism of the nucleus and cytoplasm of PGCCs resulting in rapidly changing cell morphology. Therefore, it is rational to assume that energy balance is a key aspect of the function of these cells. We have here shown that mitochondrial density augments in cisplatin-induced PGCCs along with increment in the level of intracellular ROS. Mitochondria support cancer cell survival, not only by production of ATP required for cancer cell metabolism, but also by modulation of biochemical pathways through TCA cycle byproducts^52^. Although previous concepts supported the idea that cancer cell metabolism mainly relies on glycolysis^53^, recent findings denote the fundamental effect of mitochondria on the promotion of oncogenic processes^49,54^. The increment of lactic acid in PGCCs in our metabolomics study and the rise of mitochondrial density in these cells seems paradoxical. However, the idea that PGCCs take the advantage of both aerobic and anaerobic pathways for energy production is worth to be examined in future studies.

In conclusion, PGCCs are a main source for the failure of current anti-cancer therapies. The unique metabolic profile of these cells provides opportunities for the development of novel therapeutic strategies or repositioning of current drugs. We have here shown that ZA efficiently declines the population of PGCCs through targeting the metabolic shift of these cells.

## Methods and materials

### Cell culture

This study was approved by the ethics committee of Isfahan University of Medical Sciences (IR.MUI.RESEARCH.REC.1398.719) and was conducted in accordance to the declaration of Helsinki as well as the Iranian guideline for biomedical research ethics^55^. Human foreskin fibroblasts (HFF) were isolated from a foreskin tissue obtained as a medical waste of a circumcision procedure after receiving the written informed consent. To derivate fibroblasts, the dermis was minced to small pieces and treated with 1 mg/ml collagenase A (Sigma, Germany) for 1 h at 37 °C. The digested tissue was washed with phosphate-buffered saline (PBS; Gibco, USA) and then cultured in Dulbecco’s modified Eagle's medium (DMEM) containing 10% fetal bovine serum (FBS; Gibco, USA) and 1% Penicillin–Streptomycin (Bioidea, Iran) and incubated at 37 °C and 5% CO2. The debris and non-adherent cells were discarded after 24 h and the attached cells were cultivated. Mouse bone marrow mesenchymal stem cells (MSCs) were isolated and cultured as described in our previous study^40^. Bladder cancer cell line 5637 was purchased from the Pasteur Institute of Iran and were expanded in the above-mentioned culture conditions.

To determine the optimum dose of cisplatin (Mylan, France) which induces the highest rate of polyploid cells, the cells were treated with 3, 6, 13, 18, 50, 70, and 100 μM of the drug for 72 h and then washed with PBS. After 7 days recovery period, the cells were treated with ZA (Ronak Pharmaceutical Co., Iran) dissolved in PBS at varying doses of 10, 50, 100, 300, and 600 μM for 72 h followed by twice PBS wash. After 24 h, the measurements described below were performed.

### Cell microscopy

For light microscopy (Motic, Spain), nuclei were stained with haematoxylin for 5 min and then rinsed twice with PBS immediately. Also, for fluorescent microscopy, cell membranes were stained with 0.2% Cell Tracker CM-Dil dye (Thermo Fisher Scientific, USA) for 3 min at 37 °C. To evaluate genome content by fluorescent staining, the cells were fixed with paraformaldehyde 4% for 20 min and washed twice with PBS. Subsequently, the cells were permeabilized by 0.04% Triton X-100 and stained with 0.1% 40,6-diamidino-2-phenylindole dihydrochloride (DAPI) (Sigma, Germany). Fluorescent microscopy and imaging were performed using Nikon Eclipse Ti (Nikon, Japan).

### DNA content analysis

The cells were fixed with 70% ethanol and kept at − 20 °C overnight. Subsequently, the fixed cells were treated with propidium iodide (PI; Sigma, Germany)/Triton X-100 (Cytomatin gene, Iran)/ RNase A (Sigma, Germany) solution for 30 min and were assessed by FACSCalibur instrument (BD Biosciences, USA). Flow cytometry results were analyzed using flowJo software version 10.6.2 (BD Biosciences).

### Reactive oxygen species (ROS) measurement

ROS assay kit (KiaZist, Iran) was used according to the manufacturer’s instructions. Briefly, 20 μM DCFDA reagent was added to trypsinized cells and after incubation for 45 min at 37 °C, the dye intensity was evaluated at Ex/Em = 485/525 nm by FACSCalibur instrument and the results were analyzed using flowJo software.

### Oil Red O staining

The cells were fixed with paraformaldehyde 4% for 20 min and then were washed twice with PBS. Subsequently, the cells were stained with Oil Red O staining solution for 1 min at room temperature and then were properly washed with PBS. For the quantification of lipid content, images were analyzed with Image J software (NIH, USA).

### Mitochondrial staining

Mitochondria were stained with 100 nM BioTracker 488 Green (Merck, USA) in the serum-free cell culture medium for 30 min at 37 °C. Dye intensity was measured at Ex/Em = 485/525 nm by FACSCalibur.

### Determination of total cholesterol and triglycerides content

Extraction of cell lipids was performed according to the Folch method^56^. Briefly, 3 ml ice-cold chloroform/methanol (1:2 v/v) was added to 10^5^ cells and mixed vigorously. After 5 min of incubation at room temperature, the cell suspension was centrifuged in 1000 g for 2 min at 4 °C. The lower phase containing lipids was recovered and dried with a mild flow of nitrogen gas. The dried residue was dissolved in 100 μl isopropanol, that was used for the measurement of total cholesterol and triglyceride contents using specific commercial enzymatic assays (Pars-Azmoon, Iran) according to the manufacturer’s instruction. Briefly, 1 ml of the kit reagent was added to lipid extract, dissolved in isopropanol and after 20 min of incubation at room temperature, cholesterol concentration was determined by reading the absorbance at 540 nm using Microplate readers (Bio-Rad, USA).

### gas chromatography-mass spectrometry (GC–MS)

To determine the profile of metabolites, 10^6^ bladder cancer cells before and after cisplatin treatment were trypsinized and counted with a hemocytometer. Then, ice-cold NaCl 0.9% was added to the cells suspended in DMED + 10% FBS with a 5 to 1 volume ratio. This suspension was mixed by gentle inversion and then centrifuged at 1000 g for 1 min. The supernatant was removed and 500 μl ice-cold HPLC grade methanol was added and vortexed vigorously for 30 s. The mix was frozen in liquid nitrogen and rapidly thawed at 37 °C and then centrifuged in 800 g for 1 min at 4 °C. After centrifugation, the supernatant was collected in a cryotube and kept at − 80 °C. The remaining pellet was mixed with ice-cold methanol and supernatant was added to the stored extract at − 80 °C two additional times. Subsequently, a mixture of 500 μl cold HPLC grade chloroform and 250 μl HPLC grade water was added to the pellet remained from previous the step. After freeze–thaw and centrifugation (1000 g, 3 min), the lower phase was gently collected and added to the stored extracts at − 80 °C. The remaining liquid was centrifuged for 1500 g for 1 min at 4 °C and the upper phase was gathered and mixed with previous extracts. This total extract was dried with a mild nitrogen gas flow. For derivatization, the residue was dissolved in 20 μl methoxyamine (Thermo Fisher Scientific, USA) and was heated for 90 min at 30 °C. Next, 50 μl of N, O-bis (trimethylsilyl) tri- fluoroacetamide (BSTFA; Merck, Germany)/ Chlorotrimethylsilane (TMS; Sigma, USA) (49:1 v/v) was added to the samples and were heated at 70 °C for 60 min. The resultants were cooled at room temperature and were applied to the GC–MS instrument (Agilent Technologies, USA). The GC–MS results were analyzed via Mass Hunter software (Agilent Technologies).

### Statistical analysis

Statistical analyses for all experiments were performed using unpaired Mann–Whitney test with a significance level of *p-*value < 0.05.

## Supplementary Information

Supplementary Information.

## Data Availability

All data generated are included in the manuscript.
