# Supplementary material for: Zoledronic acid targets chemo-resistant polyploid giant cancer cells
Source: Sci Rep. 2023 Jan 9;13:419. doi: 10.1038/s41598-022-27090-1 (PMC9829701; doi:10.1038/s41598-022-27090-1)
Supplement: Supplementary file 1 — Supplementary Information. [file 41598_2022_27090_MOESM1_ESM.docx]

**Supplementary information (Figures and Legends)**


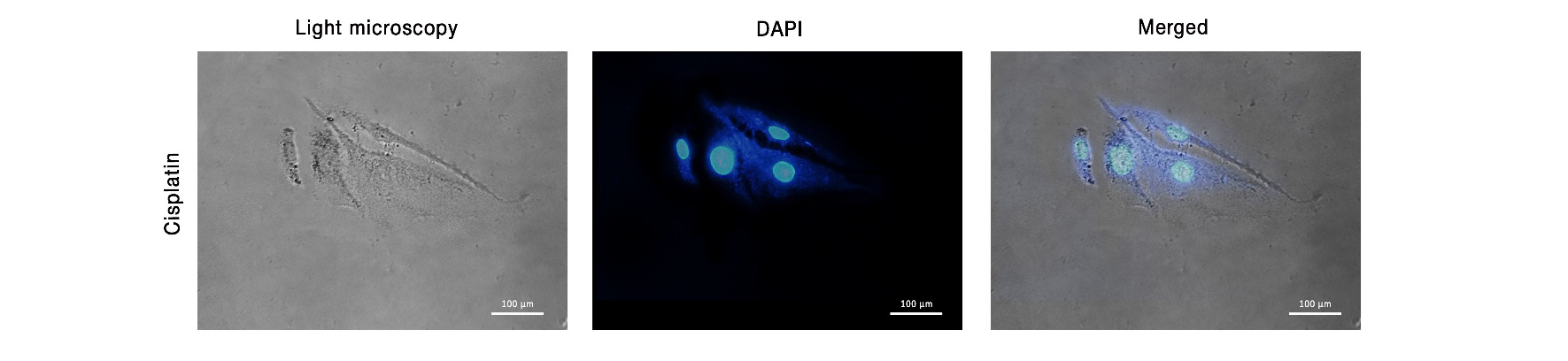
**Supplementary Figure 1.** PGCCs originate in daughter cells through bursting**.** Although budding is a common way of generating daughter cells by PGCCs, we occasionally observed that a PGCC break-down into several smaller cells by bursting. Nuclei are stained with DAPI.


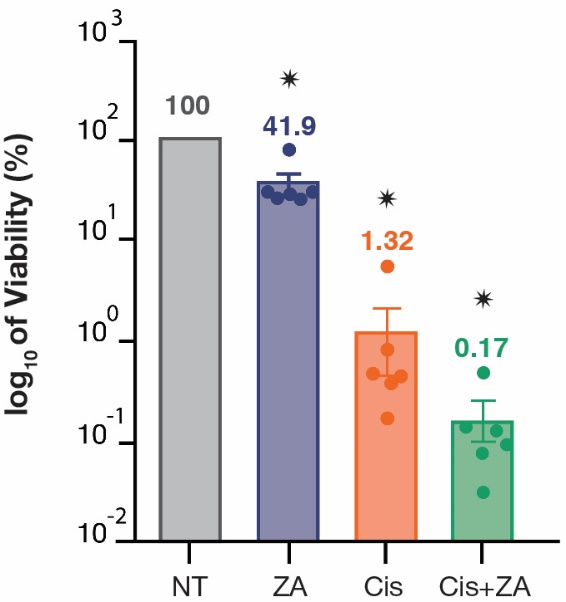


**Supplementary Figure 2**. **Zoledronic acid declines the viability of PGCCs surviving after cisplatin treatment.** Measurement of cell viability by trypan blue staining indicates the reduction of cisplatin-survived PGCCs; * p-value < 0.05


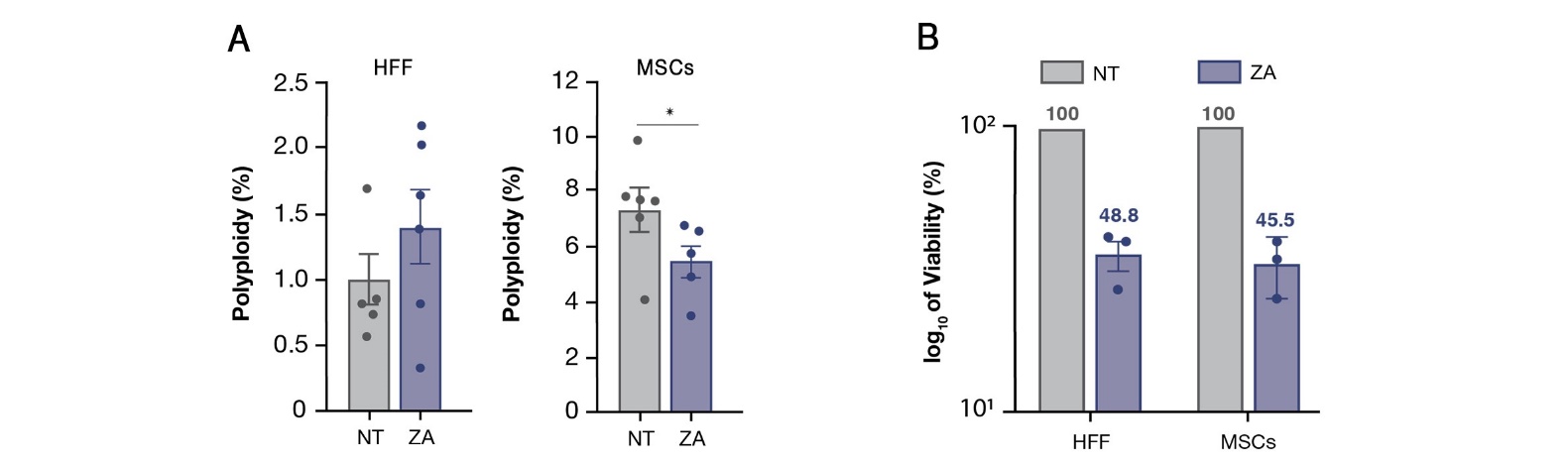


**Supplementary Figure 3**. **The effects of Zoledronic acid on non-cancerous cells.** (A) Zoledronic acid at the dose of 50 μM has an insignificant effect on the polyploidy of HFF population. The polyploidy of MSCs slightly declined post ZA administration; * p-value = 0.05 (B) The viability of HFF and MSCs, post-administration with 50 μM Zoledronic acid, was evaluated by Trypan Blue staining. Error bars: mean ± SEM.


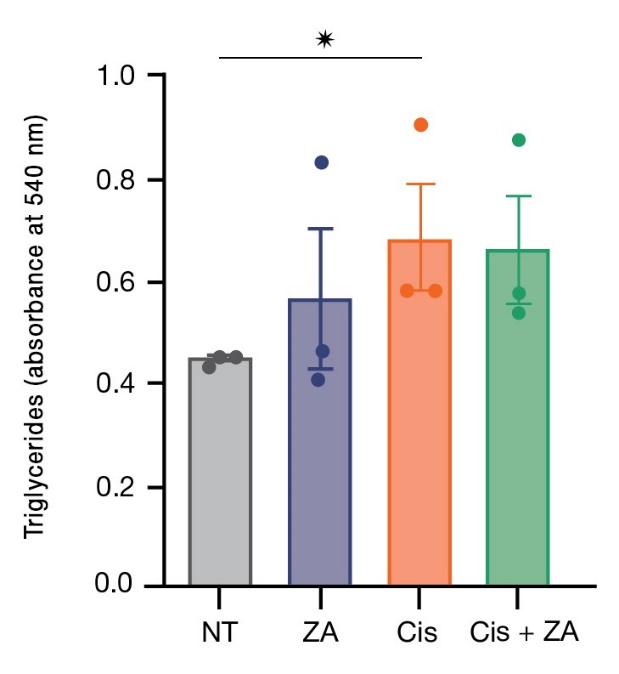


**Supplementary Figure 4. Triglycerides content.** An increment of total triglycerides was detected in the PGCC-enriched population post-cisplatin treatment. * p-value < 0.05; error bars: mean ± SEM.


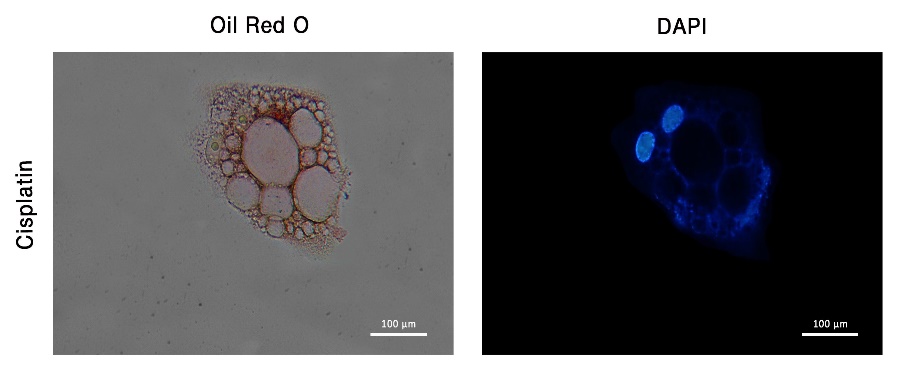


**Supplementary Figure 5** Cytoplasmic vacuoles have distinct content from nuclei and lipid droplets. Cisplatin-induced PGCCs were stained with Oil Red O and DAPI, none of which could stain the cytoplasmic vacuoles augmented after cisplatin treatment.
